# Subcellular Localization of p44/WDR77 Determines Proliferation and Differentiation of Prostate Epithelial Cells

**DOI:** 10.1371/journal.pone.0049173

**Published:** 2012-11-08

**Authors:** Shen Gao, Zhengxin Wang

**Affiliations:** Department of Cancer Biology, The University of Texas MD Anderson Cancer Center, Houston, Texas, United States of America; Southern Illinois University School of Medicine, United States of America

## Abstract

The molecular mechanism that controls the proliferation and differentiation of prostate epithelial cells is currently unknown. We previously identified a 44-kDa protein (p44/wdr77) as an androgen receptor-interacting protein that regulates a set of androgen receptor target genes in prostate epithelial cells and prostate cancer. In this study, we found that p44 localizes in the cytoplasm of prostate epithelial cells at the early stage of prostate development when cells are proliferating, and its nuclear translocation is associated with cellular and functional differentiation in adult prostate tissue. We further demonstrated that cytoplasmic p44 protein is essential for proliferation of prostate epithelial cells, whereas nuclear p44 is required for cell differentiation and prostate- specific protein secretion. These studies suggest a novel mechanism by which proliferation and differentiation of prostate epithelial cells are controlled by p44’s location in the cell.

## Introduction

During development of a multicellular organism, cells proliferate for a defined length of time before they begin functional differentiation [1], [2], [3]. Precise regulation of terminal cell division is needed to ensure production of proper numbers of differentiated cells at the appropriate time. Development of the prostate gland is a dynamic process in which epithelial cells proliferate and then functionally differentiate [4], [5]. Androgen signaling through the androgen receptor (AR) induces the growth of the prostate epithelium in early development [6] and is required later for the production of prostate-secreted proteins [7]. Other factors (NKX3.1, Hox13, FoxA, Shh, Sox9, Fgf7/10, Wnt5a, Bmp4/7, and Notch1) specifically affect ductal morphology, budding, and branching of epithelium during mouse prostate development [5]. However, the molecular mechanism that determines the timing of prostate epithelial proliferation and differentiation is currently unknown.

After puberty, a man’s serum testosterone level peaks, and his prostate gland matures [8]. Serum testosterone levels then decrease with age by an average of 0.8% per year while the prostate continues to grow [9], [10], [11]. The age-related proliferation of prostate epithelial cells is a critical step leading to prostatic intraepithelial neoplasia (PIN) and prostate cancer [10], [11], [12], [13], [14], [15]. Very little is known about what regulates this age- related growth of the prostate gland. Therefore, unraveling the molecular mechanism that controls prostate epithelial proliferation and differentiation is an important goal for understanding not only prostate development but also prostate tumorigenesis.

To search for factors that regulate the AR functions, we previously purified and cloned a novel AR-interacting protein (p44) [16] that regulates expression of a subset of AR-target genes in the prostate and prostate cancer [16], [17], [18], [19].

This protein is designated as WD40 Repeat Domain 77 (WDR77) in the NCBI Gene Bank (http://www.ncbi.nlm.nih.gov/homologene/?term=WDR77).

Human p44 is composed of 342 amino acid residues and 7 putative WD-40 repeats. The protein sequence of p44 is identical to that of a component (MEP50) of the methylosome [20] and a subunit (WD45) of the SMN complex [21]. The methylosome complex contains PRMT5, MEP50, pICln, and Sm proteins and mediates the assembly of spliceosomal snRNP [20], [22]. SMN, the protein involved in spinal muscular atrophy, is part of a complex containing the Sm proteins, WD45, and PRMT5. The SMN complex is necessary and sufficient for assembly of UsnRNA [23], [24]. Although biochemically identified in the methylosome and SMN complex, the functional role of MEP50/WD45 in the splicing process has not been proven *in vivo*.

We found the p44 immunostaining signal was strong in the nuclei of epithelial cells in normal prostate tissue but absent in the stromal cells [18]. In contrast, the nuclear staining in tumors is significantly lower, while immunostaining in the cytoplasm is strong. Similarly, p44 has been found to localize to the cytoplasm of prostate cancer cell lines LNCaP, 22RV1, PC3, and DU145 [25]. When p44 was selectively expressed in the nucleus by fusing a strong nuclear localization signal (NLS) to its N-terminus, the nuclear p44 strongly inhibited the growth of prostate cancer cells in tissue culture and in prostate tumors in nude mice by arresting the cell cycle at the G1/G0 phase transition [18], [19]. Prostate epithelium that lacked p44 did not fully differentiate and was deficient in secretory protein production [17], indicating that nuclear p44 is required for differentiation and functionality of the prostate. More recently, we also found that p44 is essential and sufficient for proliferation of lung epithelial cells, and loss of p44 expression led to the differentiation of lung epithelial cells [26]. However, p44 is not required for differentiation of lung epithelial cells.

In this report, we demonstrate that cytoplasmic p44 is essential for prostate epithelial cell growth, whereas nuclear p44 promotes cell differentiation and prostate secretory protein expression. This research reveals a novel mechanism by which the proliferation and differentiation of prostate epithelial cells are determined.

## Results

### P44 Nuclear Translocation is Associated with Functional Differentiation and Growth Arrest in Prostate Epithelial Cells

**Figure 1:**
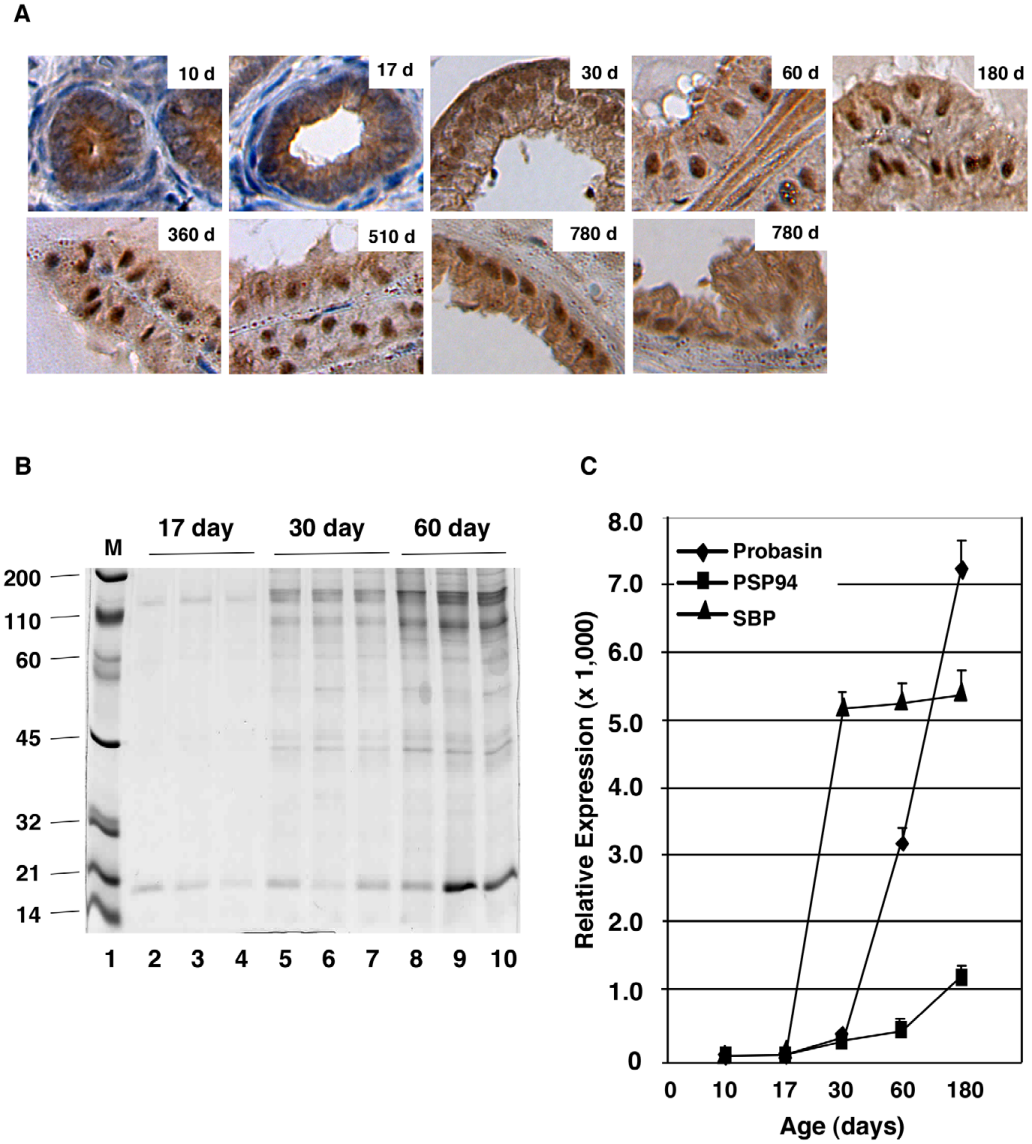
P44 nuclear translocation is associated with functional differentiation of the prostate gland. (A) P44 nuclear translocation during prostate development. P44 immunostaining (in brown) in epithelial cells in the dorsal lateral prostates of mice at various ages. (B) Analysis of secretory proteins from prostate glands of mice at 17, 30, and 60 days old. Protein secretions were resolved on a 5–20% SDS-polyacrylamide gradient gel and the gel was stained with Coomassie Brilliant Blue R-250. An equal volume (5 µl of prostate secretions was loaded onto each lane. The migration of molecular weight markers is indicated in lane 1. (C) Quantitative RT-PCR analysis of mRNA expression of three prostate-secreted proteins. Total RNAs were isolated from whole prostate glands, and amounts of three (probasin, PSP94, and SBP) mRNAs were measured by real-time PCR. Relative expression indicates specific mRNA levels divided by β-actin mRNA levels.

Previous work showed that p44 localizes in the cytoplasm of prostate epithelial cells during the early (<28 days) stage of mouse development and in the nucleus in the fully developed prostate gland (>45 days) [25]. To further characterize these findings, we studied p44 subcellular localization in dorsal lateral prostate (DLP) epithelial cells of mice up to 780 days old. Consistent with previous publications [5], this study found p44 (p44 shown in brown; nucleus shown in blue) localized to the cytoplasm of prostate epithelial cells in mice that were 10 and 17 days old (Fig. 1A). The p44 nuclear translocation was evident in prostate epithelial cells of mice at 30 days after birth, and there was very little cytoplasmic p44 by 60 days (Fig. 1A). Strong p44 nuclear signals remained in the adult prostate of mice up to 780 days old. However, we observed increased cytoplasmic p44 protein levels in epithelial cells of mice 780 days old, and the distinct p44 nuclear staining disappeared in the hyperplasia region (Fig. 1A, bottom right panel).

Antibodies to cytoskeletal keratin proteins have been used to study prostate cell differentiation [27], [28]. Those studies demonstrated that changes in the levels of differentiation are accompanied by a distinct transition in the expression profile of individual cytokeratins (CKs). Epithelial cells in the adult prostate can be characterized as basal cells (CK5^++^/CK18^−^), luminal cells (CK5^−/^CK18^++^), and intermediate cells (CK5^+^/CK18^+^). In murine prostate epithelium, p44 nuclear translocation is associated with increased expression of CK18 and decreased expression of CK5 [28]. Production and secretion of prostatic proteins are the main physiological functions of the prostate gland [4], [28], [29], [30]. We collected prostate secretory proteins from mice at the ages of 17, 30, and 60 days. Amounts of prostate-secreted proteins were significantly higher in mice at the ages of 30 (16.3±0.9 mg/ml) and 60 (26.7±1.0 mg/ml) days than in mice at the age of 17 days (4.8±0.8 mg/ml). Sodium dodecyl sulfate polyacrylamide gel electrophoresis (SDS-PAGE) analysis confirmed this observation (Fig. 1B). To further characterize prostate differentiation, we examined the expression of mRNAs of three prostate-secreted proteins (probasin, PSP94, and SBP) [31] in the mouse whole prostate gland at ages 10, 17, 30, 60, and 180 days. Low levels of all three mRNAs were noted at 10 and 17 days old, and a dramatic increase in these mRNAs was observed at 30 days and thereafter (Fig. 1C). Thus, we confirmed that p44 nuclear translocation is associated with the functional differentiation of the prostate epithelium.

**Figure 2:**
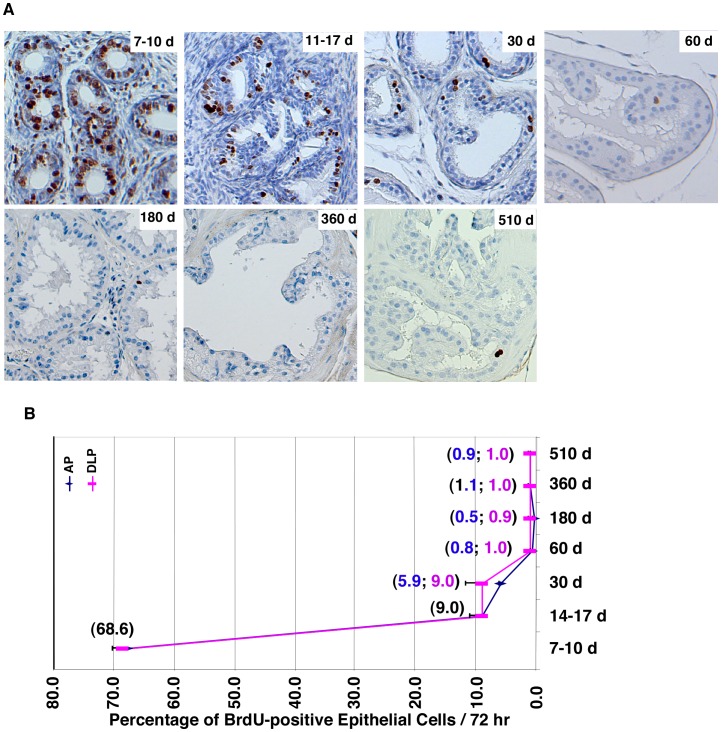
P44 nuclear translocation is associated with the decrease in cell proliferation during prostate development. (A) Proliferation rate of prostate epithelial cells is decreased during mouse development. BrdU was injected into mice, and 3 days later, inmmunostaining with anti-BrdU antibody was performed on the anterior (AP) and dorsal lateral (DLP) prostates to identify BrdU-positive (proliferating) prostate epithelial cells (in brown). (B) The quantitative data of proliferative epithelial cells in the AP or DLP prostate are shown. Numbers in parentheses indicate the percentages of BrdU-positive epithelial cells. One-way ANOVA analysis: F = 873.4, p<0.0001, R square = 0.9976. The p values of unpaired Student’s t-Test of data between 7–10 d and 14–17 d and between 30 d and 60 d are <0.0001 and 0.0060, respectively.

To examine cell proliferation, we treated mice with a bromodeoxyuridine (BrdU) pulse for 3 days to label proliferating cells. DLP sections were prepared, and cells that had BrdU incorporation (i.e., cells in S-phase) were identified by immunohistochemistry with an anti-BrdU antibody (Fig. 2A). Proliferating epithelial cells were quantified (Fig. 2B), and this quantification showed that the proliferation rate of epithelial cells was dramatically decreased during mouse development between 7 and 60 days after birth. The proliferation rate reached a low level (0.8–1.0%) in mice 60 days old and stayed at this level until 510 days. Thus, there is a good correlation between p44 nuclear translocation and decreased proliferation rate in prostate epithelial cells.

### Cytoplasmic p44 is Essential for the Growth of Prostate Epithelial Cells

**Figure 3:**
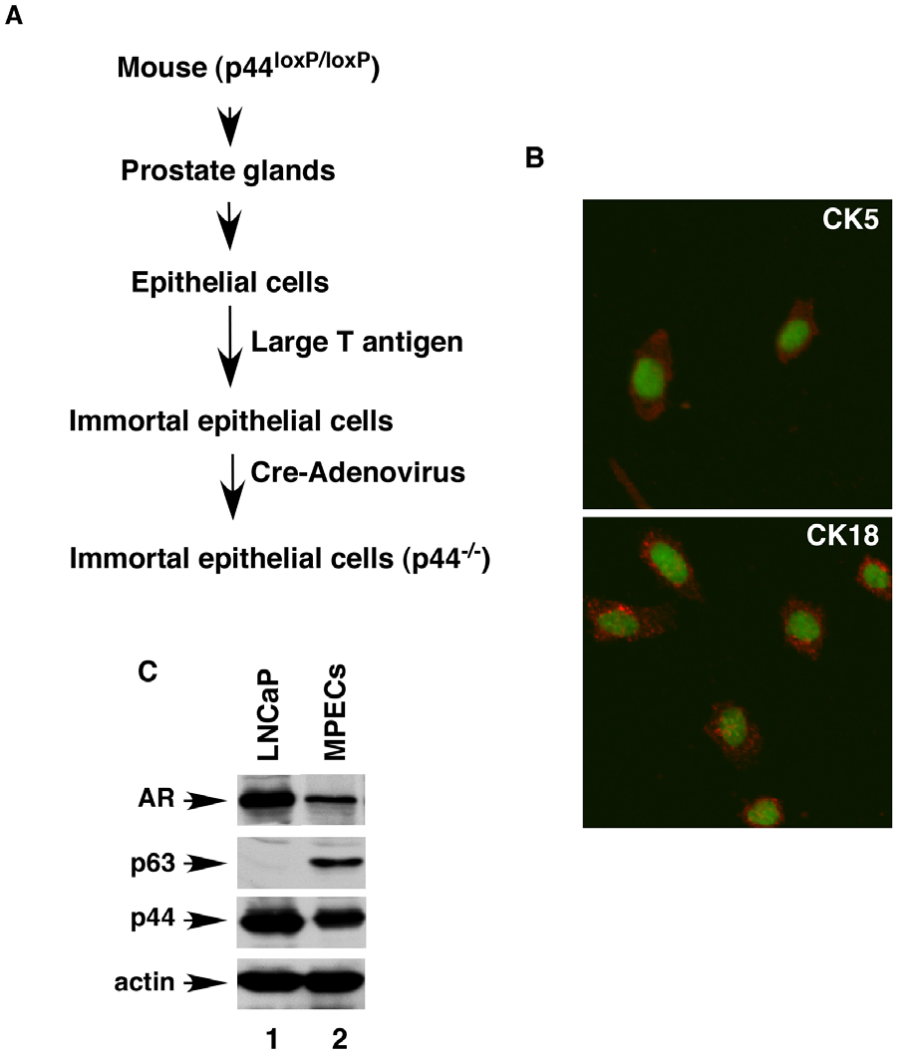
The Cre-mediated deletion of the *p44* gene in prostate epithelial cells. (A) A diagram illustrating the procedure to generate *p44-null* (p44^−/−^) epithelial cells from the mouse whole prostate. (B) Epithelial cells were stained for CK5 and CK18 as indicated. The nucleus was stained with Sytox green. (C) Western blot analysis of whole cell lysates (10 µg) made from LNCaP (lane 1) or prostate epithelial (lane 2) cells analyzed with anti- AR, -p63, -p44, or -actin antibody as indicated.

**Figure 4:**
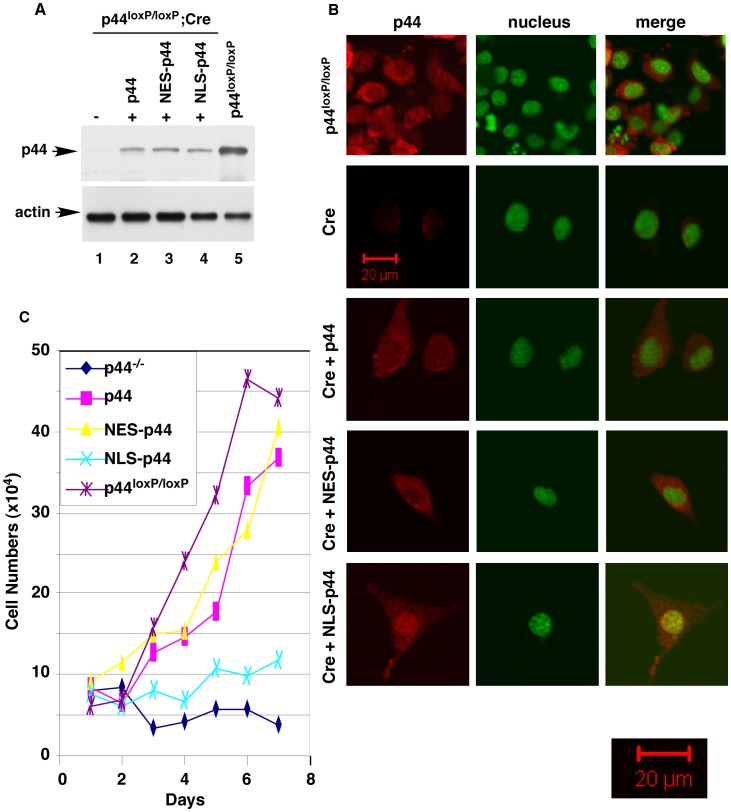
Cytoplasmic p44 is essential for growth of prostate epithelial cells. (A) Expression of p44, NES-p44, or NLS-p44 in the *p44-null* prostate epithelial cells. Western blot analysis of whole cell lysates (10 µg) made from *p44-null* prostate epithelial cells infected with control lentivirus (lane 1) or lentivirus expressing p44 (lane 2), NES-p44 (lane 3), or NLS-p44 (lane 4), as indicated. The p44 wild-type (*p^44loxP/loxp^*) epithelial cells infected with control lentivirus were used as the control (lane 5). (B) The subcellular localization of p44, NES-p44, and NLS-p44 in prostate epithelial cells. Immunostaining of p44 in p44 wild-type (*p^44loxP/loxp^*) epithelial cells (top panels), in *p44-null* prostate epithelial cells infected with control lentivirus (2^nd^ panel) or lentivirus expressing p44 (3^rd^ panel), NES-p44 (4^th^ panel), or NLS-p44 (bottom panel), as indicated. P44 and the nucleus were stained in red and green, respectively. The left pictures are a merger of p44 and nuclear staining. (C) Cytoplasmic p44 fully restored growth arrest of prostate epithelial cells induced by loss of p44. Growth curves of *p44-null* prostate epithelial cells infected with control lentivirus or lentivirus expressing p44, NES-p44, or NLS-p44. One-way ANOVA analysis: F = 43.00, p<0.0001, R square = 0.9485. The p value of unpaired Student’s t-Test of data between p44^loxP/loxP^ and NES-p44, p44, or p44^−/−^ epithelial cells are 0.3352, 0.2873, or 0.0003.

Epithelial cells were isolated from the whole prostate glands of the mice carrying a floxed (loxP-flanked) *p44* gene locus (*p44^loxp/loxp^*) at the age of 21 days [18] and then immortalized with large T antigen (Fig. 3A). The immortalized mouse prostate epithelial cells (MPECs) expressed CK5, CK18 (Fig. 3B), AR, p63, and p44 (Fig. 3C). MPECs were then infected with control adenovirus (Ad-GFP) or adenovirus harboring the Cre recombinase (Ad-Cre) to delete the *p44* gene. Western blotting and immunostaining indicated loss of p44 expression in MPECs upon infection with Ad-Cre (Fig. 4A, lane 1 versus lane 5; Fig. 4B, 1^st^ versus 2^nd^ panels). Expression of p44 could be partially restored by infecting *p44-null* MPECs with lentivirus harboring the p44 cDNA (Fig. 4A, lane 2; Fig. 4B, 3^rd^ panel). The p44 protein mainly localized to the cytoplasm in MPECs (Fig. 4B, top and 3^rd^ panels).

Deletion of the *p44* gene completely abolished MPEC growth (Fig. 4C, blue line) and resulted in complete cell death within 2 weeks. Exogenous expression of p44 (Fig. 4A, lane 2; Fig. 4B, 3^rd^ panel) or nuclear exclusion signal-tagged p44 (NES-p44, localized in the cytoplasm) [18], [19] (Fig. 4A, lane 3; Fig. 4B, 4^th^ panel) restored growth of the *p44*- *null* MPECs (Fig. 4C, pink and yellow lines, respectively). In contrast, expression of nuclear localization signal (NLS)-tagged p44 (NLS-p44) [18], [19] (Fig. 4A, lane 4; Fig. 4B, 5^th^ panel) did not have much effect on the growth of *p44-null* MPECs (Fig. 4C, green line). Dual cytoplasmic and nuclear localization of the NLS-p44 protein in some MPECs may explain a slight increase in cell numbers of NLS-p44-expressing *p44-null* MPECs. Thus, cytoplasmic p44 is essential for growth of prostate epithelial cells.

**Figure 5:**
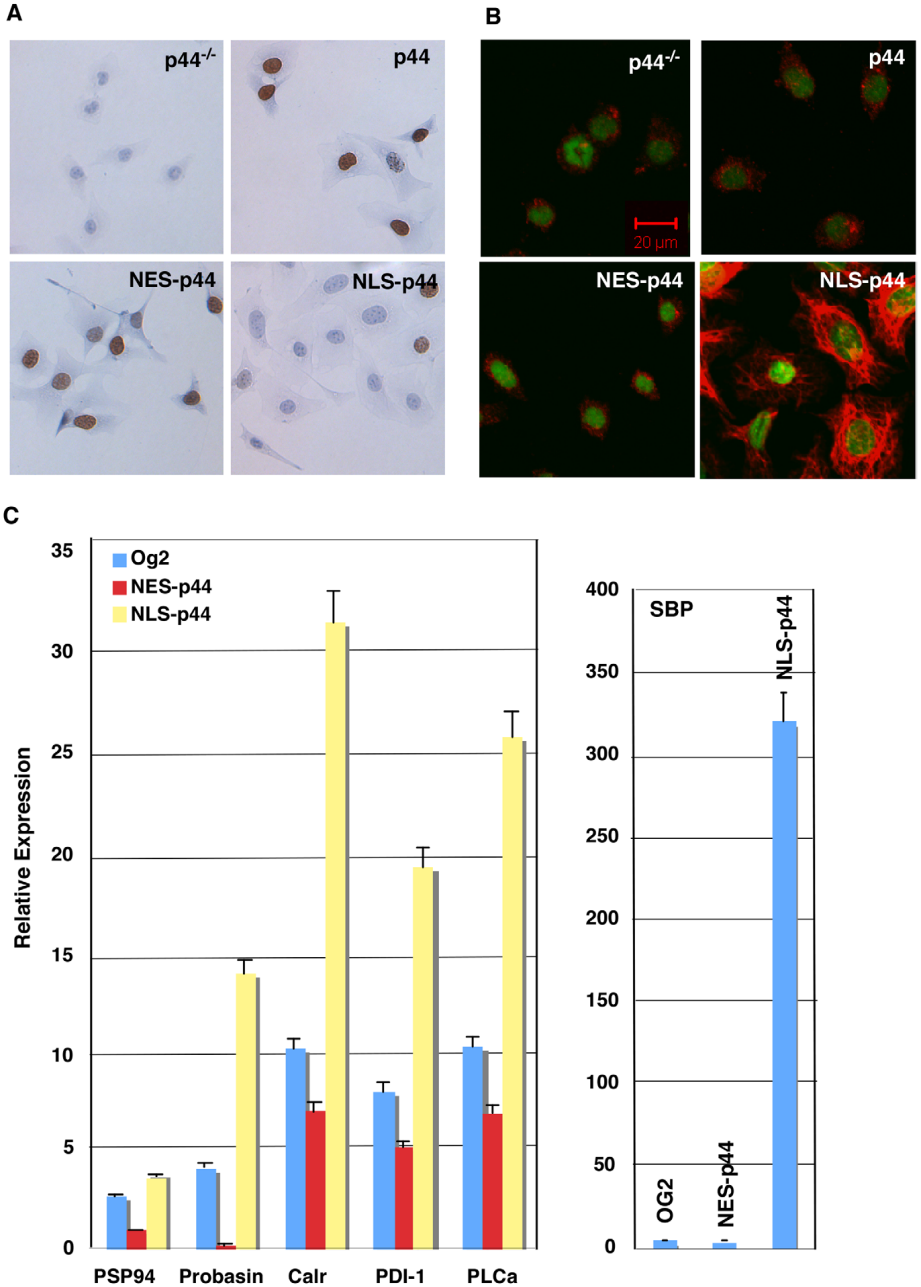
Nuclear p44 promotes differentiation of prostate epithelial cells. (A) Cytoplasmic p44 is required for proliferation of prostate epithelial cells. *P44-null* prostate epithelial cells infected with control lentivirus or lentivirus expressing p44, NES- p44, or NLS-p44 were allowed to grow in the presence of BrdU and immunostained with an anti-BrdU antibody (shown brown). (B) Nuclear p44 enhanced CK18 expression. *P44-null* prostate epithelial cells infected with control lentivirus or lentivirus expressing p44, NES-p44, or NLS-p44 were immunostained for CK18. (C) Nuclear p44 (NLS-p44) enhanced expression of prostate-secreted proteins by quantitative RT-PCR analysis of expression of prostate-secreted proteins. Total RNAs were isolated from *p44-null* prostate epithelial cells infected with control lentivirus (Og2) or lentivirus expressing p44, NES-p44, or NLS-p44 and amounts of RNAs were measured by real-time PCR. Relative expression indicated specific mRNA levels divided by β-actin mRNA levels. One-way ANOVA analysis: for PSP94 gene, F = 1,297, p<0.0001, R square = 0.9870; for probasin gene, F = 1,198, p<0.0001, R square = 0.9965; for Calr gene, F = 1,140, p<0.0001, R square = 0.9938; for PDI gene, F = 1,149, p<0.0001, R square = 0.9927; for PLCa gene, F = 1,385, p<0.0001, R square = 0.9917; and for SBP gene, F = 1,203, p<0.0001, R square = 0.9974. The p value of unpaired Student’s t-Test of data between Og2 and NES-p44 or NLS-p44 epithelial cells: 0.0103 or <0.0001 (PSP94), <0.0001 or <0.0001 (Probasin), 0.0381 or <0.0001 (Calr). 0.0005 or <0.0001 (PDI-1), 0.126 or <0.0001 (PLCa), and 0.5019 or <0.0001 (SBP).

We used a BrdU incorporation assay to measure the proliferation of MPECs (Fig. 5A). The percentages of BrdU-positive *p44^loxP/loxP^* MPECs (83% ±7%) were much higher than those of *p44-null* MPECs (2.5% ±1%), indicating that the loss of p44 expression inhibited the proliferation of MPECs. Exogenous expression of p44 (78% ±8%) or NES-p44 (82% ±6%) restored proliferation of *p44-null* MPECs, but expression of NLS-p44 only slightly enhanced it (5% ±1%). Thus, cytoplasmic p44 is essential for proliferation of prostate epithelial cells.

### Nuclear p44 Promotes Cell Differentiation

To directly test the role of nuclear p44 in MPECs, we expressed NLS-p44 to force p44 nuclear translocation in the *p44-null* MPECs (Fig. 4B, 5^th^ panel). NLS-p44 expression dramatically increased expression of CK18 (Fig. 5B) and six prostate secretory proteins (Fig. 5C). In contrast, expression of NES-p44 inhibited expression of these proteins (Fig. 5C). These data confirm that nuclear p44 is required for the differentiation and functionality of prostate epithelial cells. NLS-p44 also extended the survival time (>30 days) of the *p44-null* MPECs in tissue culture (Fig. 4C).

### Proliferation and Differentiation of Prostate Epithelial Cells in a Temperature- Dependent Manner

**Figure 6:**
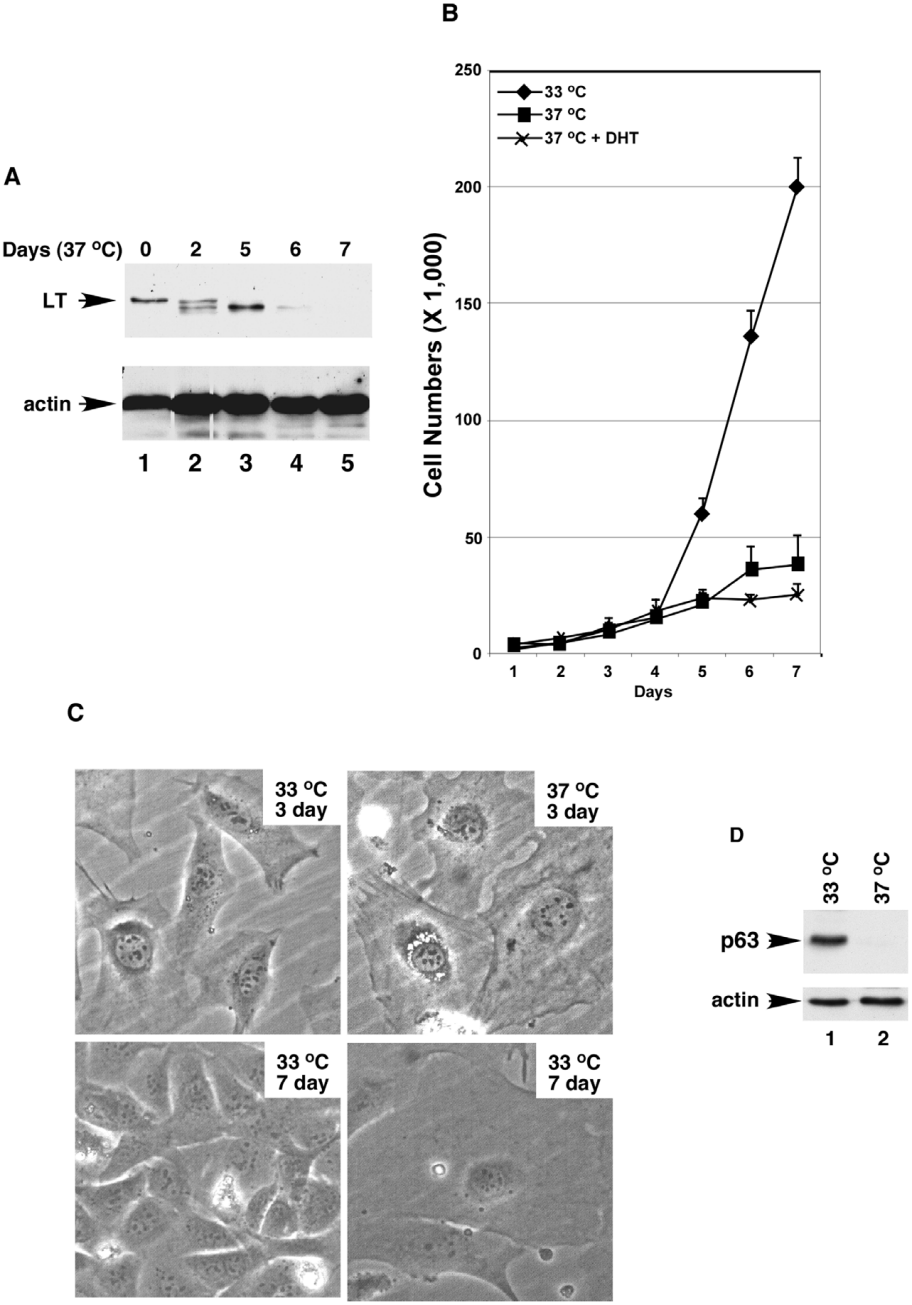
The growth and differentiation of prostate epithelial cells are temperature-dependent. ( A) Temperature-dependent inactivation of the large T antigen (LT). Western blot of whole cell lysates derived from temperature sensitive LT prostate epithelial cells (LT^ts^-ECs) grown at 33°C (lane 1) or 37°C (lanes 2–5) with anti-LTA or -actin antibody as indicated. (B) Non-permissive temperature (37°C) induced prostate epithelial cells to enter a quiescent state. Growth curves of LTA^ts^-ECs at 33 or 37°C without or with additional androgen (DHT, 10 nM). One-way ANOVA analysis: F = 83.02, p = 0.0006, R square = 0.9722. The p value of unpaired Student’s t-Test of data between prostate epithelial cells at 33°C and 37°C or 37°C plus DHT is 0.0005 or 0.004. (C) The non-permissive temperature altered the cellular structure of prostate epithelial cells. Prostate epithelial cells were cultured at 33 or 37°C for 3 or 7 days and photographed. (D) P63 expression was lost when prostate cells were grown at 37°C. Western blot analysis of whole cell lysates (10 µg) made from prostate epithelial cells grown at 33°C (lane 1) or 37°C (lane 2) with anti-p63 (top) or β-actin (bottom) antibody.

The transgenic mouse strain *H-2K^b^-tsA58*, whose tissues harbor a temperature-sensitive simian virus 40 (SV40) large tumor (LT) antigen, has been used to isolate and propagate primary cells from various organs for long-term culture [32], [33]. We used this mouse strain to generate a culture of mouse prostate epithelial cells. LT^ts^-MPECs were isolated from the whole prostate of mouse at the age of 21 days and cultured under permissive temperature (33°C), that is, a temperature at which the LT antigen was functional (Fig. 6A, lane 1); the LT^ts^-MPECs were immortal and grew normally in tissue culture with a doubling time of about 20 hr (Fig. 6B). Transfer of the cultured LT^ts^-MPECs to non-permissive temperature (37°C) resulted in the induction of a quiescent state due to inactivation of the LT antigen (Fig. 6A, lanes 2–5; Fig. 5B). Additional dihydrotestosterone (DHT) added into the medium further enhanced the induction of the quiescent state (Fig. 6B). Cell sizes increased when LTts-MPECs were grown at 37°C (Fig. 6C).

**Figure 7:**
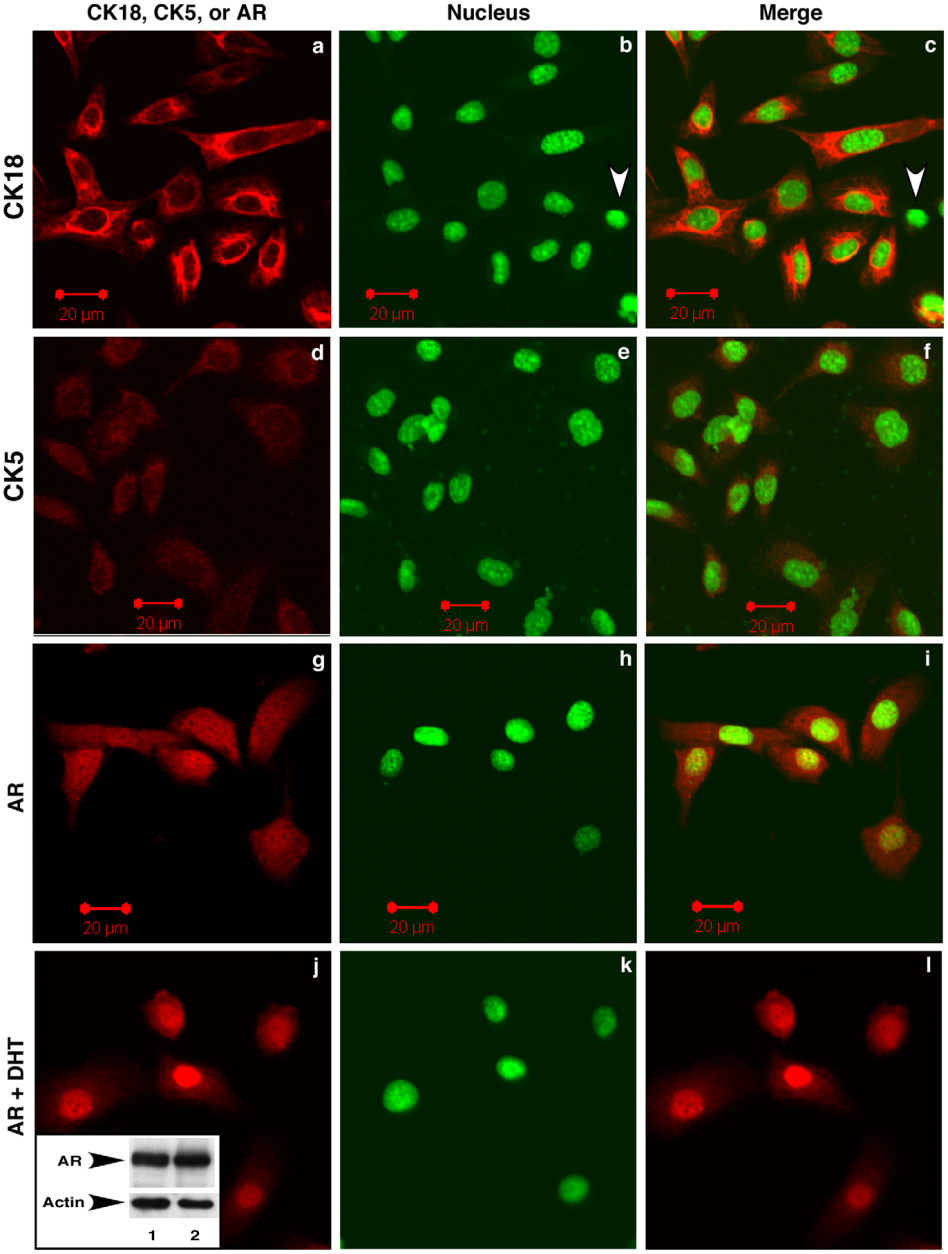
Temperature-sensitive large T antigen-immortalized prostate epithelial cells (LTA^ts^-ECs) express markers of prostate epithelial cells. LTA^ts^-ECs were grown at 33°C without (panels a-i) or with 10 nM DHT (panels j-l) and immunostained with anti-CK18, CK5, or AR antibody. Nuclei were stained with Sytox green. The inset in panel j shows AR expression in prostate epithelial cells detected by Western blotting with anti-AR antibody.

The basal cell marker p63 was expressed in LT^ts^-MPECs cultured at 33°C but not in those grown at 37°C (Fig. 6D). Almost all the LT^ts^-MPECs (>95%) expressed epithelial cell markers AR and CK18 (Fig. 7, white arrowheads in panels b and c indicate a cell without CK18 expression). Androgen-driven AR nuclear translocation was observed in LT^ts^-MPECs (Fig. 7, panels j-l versus panels g-i). The cultured LT^ts^-MPECs also expressed low levels of the basal cell marker CK5 (Fig. 7, 2^nd^ panel). Thus, the isolated LT^ts^-MPECs resembled prostate epithelial cells at the intermediate differentiation stage.

**Figure 8:**
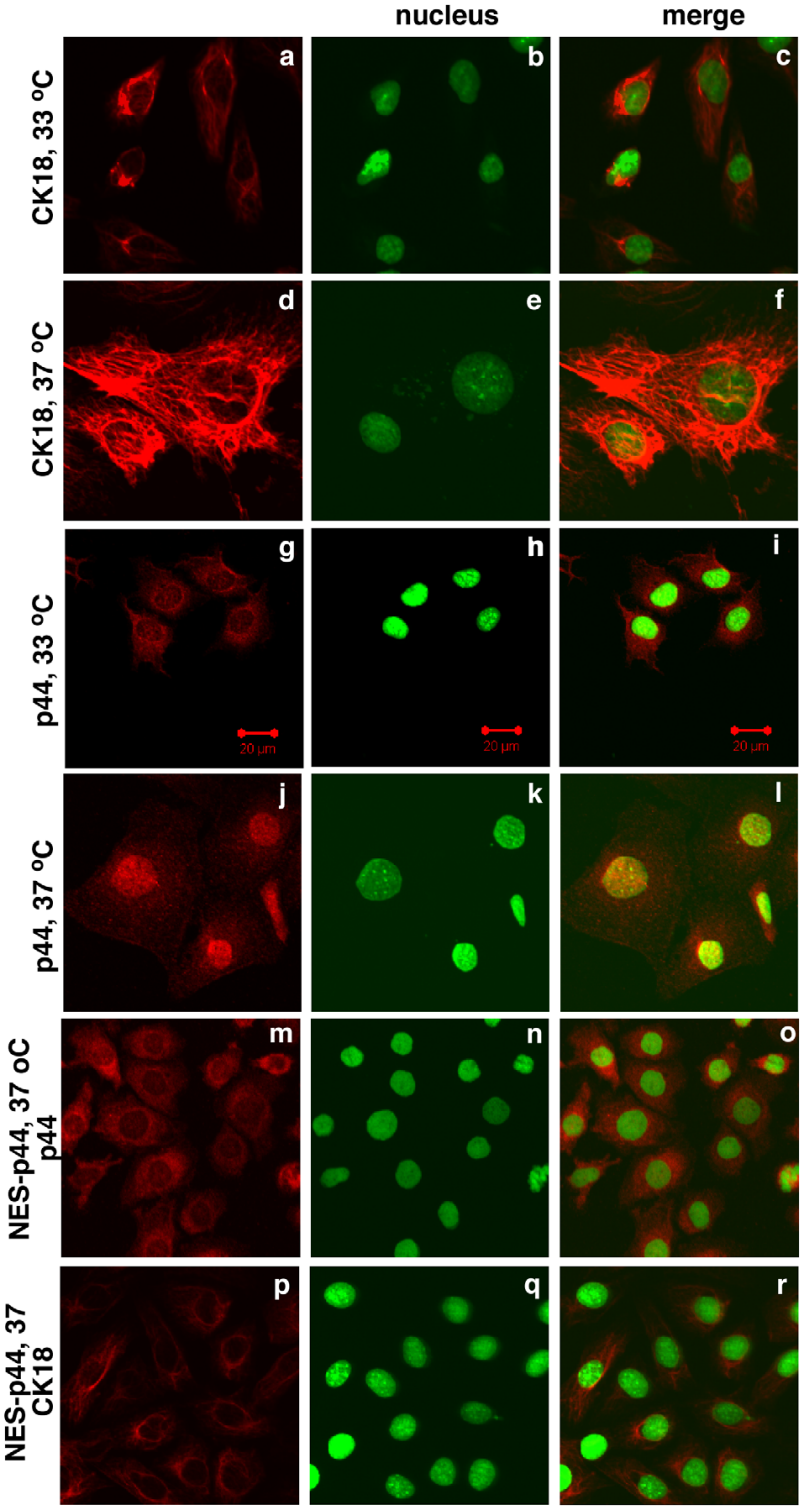
Cytoplasmic p44 inhibited the temperature-dependent differentiation of prostate epithelial cells. LT^ts^-ECs (panels a-l) or LT^ts^-ECs expressing NES-p44 (panels m-t) were grown at 33°C (1^st^ and 3^rd^ panels) or 37°C (2^nd^, 4^th^, 5^th^, and 6^th^ panels) and immunostained with anti-CK18 (1^st^, 2^nd^, and 6^th^ panels) or -p44 (3^rd^, 4^th^, and 5^th^ panels). Nuclei were stained with Sytox green.

**Figure 9:**
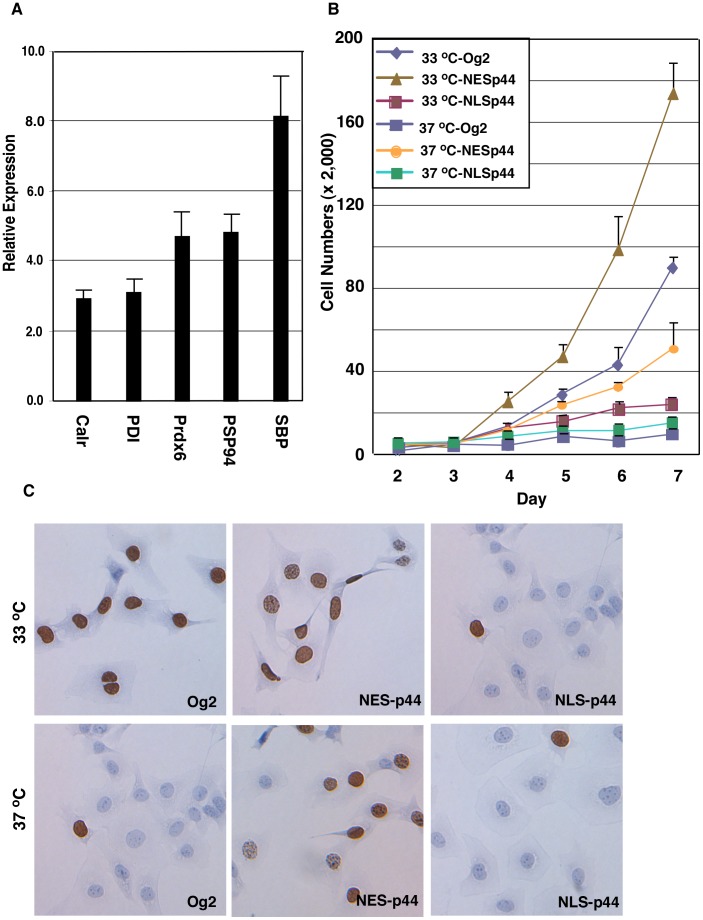
Cytoplasmic p44 promotes and nuclear p44 inhibits proliferation of prostate epithelial cells. (A) Nuclear p44 enhanced expression of prostate-secreted proteins. A, Quantitative RT-PCR analysis of expression of prostate-secreted proteins. Total RNAs were isolated from LT^ts^-ECs cultured at 33°C or 37°C for 6 days, and amounts of specific RNAs were measured by RT-PCR. Relative expression indicates specific AR expression in LTA^ts^-ECs cultured at 37°C divided by amounts of the same RNA in LTA^ts^-ECs cultured at 33°C. (B) Cytoplasmic p44 fully restored the growth inhibition of prostate epithelial cells induced by the non-permissive temperature. Growth curves of LTA^ts^-ECs infected with control lentivirus (Og2) or lentivirus expressing p44, NES-p44, or NLS-p44 and grown at 33°C or 37°C. One-way ANOVA analysis: F = 196.5, p = <0.0001, R square = 0.9861. The p value of unpaired Student’s t-Test of data between 37°C-Og2 and 33°C-NESp44, 33°C-NESp44, 37°C-NESp44, 33°C-NLSp44, or 37°C-NLSp44 is <0.0001, <0.0001, 0.0053, 0.0017, or 0.0038. (C) Nuclear p44 inhibited proliferation of prostate epithelial cells. LTA^ts^-ECs infected with control lentivirus (Og2) or lentivirus expressing p44, NES-p44, or NLS-p44 were allowed to grow in the presence of BrdU and immunostained with an anti-BrdU antibody (shown blown).

The dramatic increase in CK18 expression in LT^ts^-MPECs cultured at 37°C (Fig. 8, panels d-f) relative to those cultured at 33°C (Fig. 8, panels a-c) indicated differentiation of the LT^ts^-MPECs. Expression of five prostate secretory proteins [34] increased in LT^ts^- MPECs cultured at 37°C compared to those grown at 33°C (Fig. 9A), also indicating the functional differentiation of LT^ts^-MPECs cultured at 37°C.

### P44 Nuclear Translocation is Essential for Differentiation of MPECs

The p44 protein localized in the cytoplasm when LT^ts^-MPECs were proliferating (at 33°C) (Fig. 8, panels g-i) and in the nucleus when LT^ts^-MPECs were quiescent and differentiated (at 37°C; Fig. 8, panels j-l). Thus, similar to p44 in epithelial cells in the prostate gland, p44 nuclear translocation was associated with cell differentiation.

Similar to that in prostate cancer cells, expression of NES-p44 in LT^ts^-MPECs further enhanced the cells’ growth, whereas expression of NLS-p44 inhibited the growth of LT^ts^- MPECs cultured at 33°C (Fig. 9B). To our surprise, expression of NES-p44 promoted growth of LT^ts^-MPECs cultured at 37°C (Fig. 9B), indicating that p44 in the cytoplasm can drive the growth of quiescent epithelial cells. Expression of NES-p44 blocked temperature-induced p44 nuclear translocation (Fig. 8, panels m-o) and CK18 expression (Fig. 8, panels p-r). Thus, nuclear p44 is essential for differentiation of prostate epithelial cells.

We used a BrdU incorporation assay to measure the proliferation of LT^ts^-MPECs (Fig. 9C). The percentages of BrdU-positive LT^ts^-MPECs cultured at 33°C (83% ±8%) were significant higher than those of LT^ts^-MPECs cultured at 37°C (5% ±1%), indicating that the loss of proliferation of LT^ts^-MPECs at non-permissive temperature. Exogenous expression of NES-p44 significantly increased proliferation of LT^ts^-MPECs cultured at both 33°C (95% ±6%) and 37°C (66% ±6%). In contrast, expression of NLS-p44 inhibited proliferation (22% ±3%) of LT^ts^-MPECs cultured at 33°C. These results suggest that the quiescence that occurred at 37°C was largely due to blockage of cell proliferation induced by p44’s translocation from the cytoplasm to the nucleus.

## Discussion

We have demonstrated that cytoplasmic p44 is essential for proliferation whereas nuclear p44 is required for differentiation of MPECs. These findings suggest a novel mechanism by which p44 controls the proliferation and differentiation of epithelial cells during prostate development and tumorigenesis.

The results of the present study suggest that p44 plays essential roles in cell proliferation in the prostate. Specifically, we found that p44 cytoplasmic localization was associated with the proliferation of MPECs during development. P44 is in abundance in the cytoplasm of prostate epithelial cells harvested from mice during the early stages of development, when cells are proliferating, and in growing immortalized temperature-_sensitive lung epithelial cells (LT_^ts^_-MPECs). In contrast, p44 expression was limited in_ the nucleus of adult MPECs and of immortalized growth-arrested LT^ts^-MPECs. In addition, we found that p44 expression was sufficient to promote proliferation of LT- immortalized growth-arrested LT^ts^-MPECs at a non-permissive temperature. Finally, we found that deletion of the *p44* gene abolished the growth of MPECs, and expression of NES-p44, but not NLS-p44, completely restored this growth deficiency. These results strongly indicate that cytoplasmic p44 is essential for the growth of prostate epithelial cells.

During the development of a multicellular organism, cells proliferate for a defined length of time before they begin to differentiate functionally [1], [2], [3]. Terminal cell division must be precisely regulated to ensure that the proper numbers of differentiated cells are produced at the appropriate times. Although the control of proliferation and differentiation is highly coordinated, certain differentiation decisions are not compatible with continued proliferation. One essential component of these processes in the prostate gland is p44. The presence of p44 in the cytoplasm of prostate epithelial cells (PECs) during the early stages of prostate development is essential for their proliferation. When proper numbers of prostate epithelial cells are generated, p44 translocates into the nucleus, and proliferation stops. Nuclear p44 is an AR cofactor and drives expression of a set of AR target genes to promote PEC differentiation [17], [18]. In the prostate, p44 plays roles in proliferation or differentiation, depending on its subcellular localization. We have also found that p44 is essential for epithelial cells to progress through the G1 phase [17], [18]. The process of differentiation of primitive cells into more specialized cells involves an increasing restriction in proliferation capacity culminating in cell-cycle exit (34.35). Maintenance of cell-cycle arrest in terminally differentiated cells is important for the ultimate architecture and function of specific tissues [36], [37], [38]. Whether a cell will differentiate into one or another cell type is usually determined at the G1 phase of the cell cycle [36], [39] Consistent with these studies, loss of p44 expression inhibited the proliferation of PECs and prostate cancer cells by means of arrest at the G1 phase.

Our findings suggest that p44 also plays an important role in prostate cancer. Tumor cells do not differentiate normally and have an unlimited capacity to proliferate. It is likely that p44 exerts the same biological activities during tumorigenesis as it does during normal prostate development. We detected the cytoplasmic localization of p44 in prostate cancer samples [17], [18], which suggests that cytoplasmic p44 has an important role in the proliferation of prostate cancer cells. Indeed, p44 silencing or expression of NLS-p44 dramatically inhibited prostate cancer cell growth in tissue culture and abolished the growth of prostate tumor xenografts in nude mice [17], [18]. Thus, p44 cytoplasmic expression is also essential for prostate tumor growth. The molecular events that cause terminally differentiated PECs to re-enter the cell cycle remain unidentified; however, our findings suggest that the cytoplasmic translocation of p44 is essential to this process. Our findings also suggest that the p44 cytoplasmic translocation event is a novel target for the prevention and treatment of prostate cancer. However, the ways in which this developmental program is reactivated in prostate cancer cells remains unknown. Our current work is focused on identifying the regulatory networks controlling p44 subcellular translocation during prostate development, which may give us some clues about prostate tumorigenesis.

## Materials and Methods

### Animals

Mice were handled in accordance with the guidelines published in the National Institutes of Health Guide for the Care and Use of Laboratory Animals. The MD Anderson Institutional Animal Care and Use Committee approved all the experimental procedures used for mice. Mice were sacrificed by CO_2_.

### Mouse Prostate Preparation

Male C57BL6/J mice (n = 40) at the age of 7, 14, 30, 60, 180, 360, 510, or 780 days were injected with BrdU (0.1 mg per g of body weight per day; Sigma-Aldrich, St. Louis, MO) via intraperitoneal injection for 3 days. Mice were killed, and whole prostate glands were freed from surrounding structures and fixed by immersion in 4% paraformaldehyde in phosphate-buffered saline (PBS) overnight at 4°C. The tissues were then embedded in paraffin, and sections (4 µm) were cut and mounted on Super-frost Plus adhesion slides (Fisher, Pittsburgh, PA) for hematoxylin and eosin (H&E) and immunohistochemical staining.

### Collection of Secretions

Luminal material was collected from mouse prostate glands. The whole prostate was minced in 0.1 ml of PBS containing protease inhibitors (1 mM phenylmethane sulfonylfluoride, 10 µM leupeptin, 1.4 µM pepstatin A). The tissue was then centrifuged at 16,000×g for 2 min at 4°C, the supernatant was drawn off, and the protein concentrations were determined by using the Bradford method (Bio-Rad) with bovine serum albumin (Bio- Rad) as the standard. The supernatant was mixed with 0.1 ml of 2×SDS gel sample buffer. The samples were then heated to 100^o^C for 5 min and on a 5 to 20% SDS polyacrylamide gradient gel. The gel was stained with Coomassie blue R 250.

### Isolation and Culture of MPECs

Male *p44^loxP/loxP^* (n = 5) or *H-2K^b^-tsA58* (n = 5) mice were killed at the age of 21 days. The whole prostates were then microdissected, minced, and incubated with collagenase, pronase, and DNAse I at 37°C for 2 hr. The epithelial organoids were separated from the stromal cells and debris by iso-osmotic Percoll gradient centrifugation (500 x g, 30 min). After centrifugation, the stromal cells remained near the top of the gradient while the epithelial cell organoids banded at a higher density near the middle (ρ = 1.05−1.07 g/ml) of the gradient. Epithelial organoids were collected and washed with M199 medium. The epithelial organoids were plated onto a culture dish that had been coated with 5 µg/ml mouse laminin (Sigma-Aldrich) in keratocyte-SFM medium (GIBCO) supplemented with 2% fetal bovine serum (FBS), epidermal growth factor (EGF), and bovine pituitary extract. Upon reaching confluence, cells were re-plated to a new collagen-coated plate at a split ratio of 1∶2.

To immortalize PECs, epithelial cells were infected with lentivirus expressing LT antigen. Two days after infection, epithelial cells were split 1∶4 and selected with G418 (0.5 mg/ml) for 2 weeks. The G418-resistant cells were expanded.

### BrdU Labeling of Cultured Cells

For the BrdU incorporation assay, cells (50–70% confluent) were cultured on coverslips in the presence of BrdU (10 µM) for 8 hrs. The BrdU-labeled cells were detected employing a monoclonal anti-BrdU antibody (BD Biosciences, Franklin Lakes, NJ).

### Real-time PCR

Male C57BL6/J mice (n = 20) at the age of 7, 14, 30, or 60 days were killed, and the whole prostate glands were dissected. Total RNA was isolated using the TRIzol Reagent and reverse transcribed using the Reaction Ready First Strand cDNA Synthesis Kit (SuperArray Bioscience Corp., Frederick, MD). The cDNA products were PCR- amplified (40 cycles of 30 s at 94°C; 20 s at 55°C; 30 s at 72°C) with the RT^2^ real–time SYBR green PCR master mix and the gene-specific primer sets for mouse genes encoding prostate-secreted proteins and β-actin genes (SuperArray Bioscience Corp.) in a SmartCycler II (Cepheid, Sunnyvale, CA). Raw data processing and quantification were performed with the SmartCycler software (version 2.0C) [40].

### Immunohistochemistry

Formalin-fixed, paraffin-embedded mouse prostate sections were deparaffinized by sequential washing with xylene, graded ethanol, and PBS. Antigen retrieval was done by heating the samples in a steam cooker in 1×Target Retrieval Solution (Dako) for 30 min. After the samples were cooled and washed with PBS, endogenous peroxide was blocked with 3% hydrogen peroxidase inhibitor in PBS for 12 min. Nonspecific proteins were blocked by immersing the sections in 5% horse serum and 1% goat serum for 20 min. Slides were incubated with anti-p44 (1∶400), -CK18, -CK5, -BrdU, or -Ki67 antibodies overnight at 4°C and then with a secondary peroxidase-labeled anti-rabbit antibody (1∶500; Jackson ImmunoResearch) for 1 h at room temperature. Signal was detected by staining with 3,3′-diaminobenzidine (DAB; Phoenix Biotechnologies) substrate for 6 min and then counterstaining with Gill’s hematoxylin No. 3 (Sigma) for 20 s.

For cultured cells, cells were grown on chamber slides and fixed with cold methanol (−20°C) for 10 min. Nonspecific proteins were blocked in 4% fish gelatin in PBS for 20 min. Overnight incubation at 4°C with primary antibodies was performed followed by a 1-hr incubation with goat anti-rat Alexa 595 (1∶500; Invitrogen) at room temperature. After washes in PBS, the samples were counterstained with Sytox green (Molecular Probes) for 10 min at room temperature, mounted in Histogel (Linaris Histogel), and analyzed directly by fluorescence confocal microscopy.

### Statistical Analysis

The one-way ANOVA analysis and unpaired Student’s t-Test was performed using the GrapPad Prism 6.0 program.
